# Supplementary material for: Patterns of Intron Gain and Loss in Fungi
Source: PLoS Biol. 2004 Nov 30;2(12):e422. doi: 10.1371/journal.pbio.0020422 (PMC532390; doi:10.1371/journal.pbio.0020422)
Supplement: Table S1 — Also available at http://genes.mit.edu/NielsenEtAl/. (4.3 MB ZIP). [file pbio.0020422.st001.zip › NielsenEtAl/html/723.html]

AN5786.1.NCU07955.1.MG05135.1.FG01344.1


```
 CLUSTAL W (1.82) Multiple Sequence Alignments - Introns Inserted


Sequence 1: NCU07955.1	671 aa
Sequence 2: MG05135.1	796 aa
Sequence 3: FG01344.1	742 aa
Sequence 4: AN5786.1	707 aa
Alignment Length: 835 aa
Number Identitical Residues: 281 aa
Alignment Score (without introns) 14266


MG05135.1 	-------------MPSTSNPQLGRQLWAAIRDSIDISSVWIR---FPVRTQAARSRFHHR
NCU07955.1	--------------------------------------------------------MGHL
FG01344.1 	MAKGIPSSVIGRCLGTSSNYSPAIFASTPIRTVTELSARWPT---TPILFSNVR--LNHT
AN5786.1  	-MSGIFGRKLYTTFWKTASPFLGRGLRVTRAENLSHTASQIDSLYKSILKQYAKKLPTAP
          	  ..  .        .::.   .     .     . ::    :   .   .   .     

MG05135.1 	PEEALRSLQQSPFSSAKRSFHTSNTSRQRTTSTIYTKHSPAHIKRNCTSGSLLLLGLETP
NCU07955.1	ARQALLLRR----------------IRQNTTTTTLCKTG----KRFQSSG----------
FG01344.1 	QRQTQIWKQTVSNG-VPGTPVFPLCHAIKPQKSMRERTTPLTAQSLISQPS---------
AN5786.1  	PRKTNQITRRFASGSCLILGVSPSSTVVETGTTCAVSSEKIISHRLSPFNR---------
          	 .::    :  . .:       .     .. .:          :   .            

MG05135.1 	TSPSTQTTVTTCAGAAADSVLLASGGSSKPSISALARQAWRRNVHTGRGGQQTPRNDQPK
NCU07955.1	--------------------------------------------------------KETK
FG01344.1 	--------------------------------ALLSRSAITWQQHRFYSGRGSSSGNGPK
AN5786.1  	-----------------------------------------------WFPHVLSRNVQTR
          	                                                     . .  .:

MG05135.1 	TSN--DDVKSSTGKDGVSATGSRDGPSSVKSSDQANARPDLKSEANPAPHEETLTESMSK
NCU07955.1	GDH--DETKAEAKSSTISPQQKSEQPQQPPNTETASG-----SGSQPTPAHESIADSMSK
FG01344.1 	CSCGKDISDIPPTPARTTEDLKKVKPSTLSETSKDATNQTTKSGDQVPAGTNSEPVSYMS
AN5786.1  	GFQTNKDDVSRAGARTDAADRAGVKNVETEGKHQKSE----EQSFRPGEKHEPAPPNRPL
          	    ..     .     :             .         ..  .     :. . .   

MG05135.1 	YLHMPKMP---HRPTKEELLAAANGFFQRLKVRFKWLSIRSMRPYNADEWGAFVSWFMLG
NCU07955.1	YLHLPHLPHLPHRPTKEELLAAASGFWSRLKVRFKWFSIRSMRPWNADEWGAFVSFFLFG
FG01344.1 	YLHLPRMP---HRPTKDELLEAANGFWQRLKVRLKWISIRSMRPWNIDEWGAFVSWFLFG
AN5786.1  	IHRLPHMP-HLHRPTKEELLAAATGFWSRLKVRFKWFSIRSVRPFNLDEIAALFSWVILG
          	  ::*::*   *****:*** **.**:.*****:**:****:**:* ** .*:.*:.::*

MG05135.1 	HLVWILVGTTTFFSLIILFVNTVFAQE1TLAKWIGDYLTQSAGVTVVFESAIVPKWKDGV
NCU07955.1	HLVWILVGTTTFFSLIILFINTVFAQE~TLAKWIGDYLTQSAGLTVVFESAIVPKWGDGV
FG01344.1 	HLAWIIVGTTTFFSLIILSINTVVAQE1TLAQWVGDYLTQSAGVTVVFESAIVPKWRDNV
AN5786.1  	HIVWIVLGTTTFFSLLILAINTVFAQE1TLAGWIGNYLTKSSGVKVVFESAIVPKWRDGV
          	*:.**::********:** :***.*** *** *:*:***:*:*:.*********** *.*

MG05135.1 	ITFRNVFISRRPGQ-SKSSVKKGSSTSAAAVAAARQTQ--------PSE-----EDDGNY
NCU07955.1	ITFRNVFVSRRPGQ-PKSSVKKGSSEAAAEAAAAKQAA--------EQEGTAVEEDDGNY
FG01344.1 	ISFRNVFVSRRPGQGNVSSVSKGSSDAAAEAAAGRKAE--------APEV----EDDGNY
AN5786.1  	ITFKNVFVSRRPGQ-GTGHVSKGSSKTAAAAAAAAHLGDQSGADLLEQRISSEEEEDTNY
          	*:*:***:******   . *.**** :** .**. :  ..:.:.    . :: .*:* **

MG05135.1 	TQFDVTINTVNVTLSFYKWWNGTGLLKDVEVKGVRGVLDRTSVRWSDGPHDPFSYRHVHQ
NCU07955.1	TQFDVTIDTVNVALSFLKWWNGKGLLKDVEIKGVRGVVDRTSVVWGNEDLNPLDFRQEHN
FG01344.1 	TQFDITLSTVNVTLSFLNWWNGKGLLKDVEIKGVRGVLDRTSVTWPVEEVDPLSYRHKHQ
AN5786.1  	TQFDLSIETVNVTLSFGRWINGKGLLRDVEVKGVRGVVDRRSVFWPDEPRDPKSYRHEHN
          	****:::.****:*** .* **.***:***:******:** ** *     :* .:*: *:

MG05135.1 	PGDFEIEHFKLEDLLLTVHQPGGFRPFP~VSIYSCELPQLRKQWLYYDFLNANHCSGSFD
NCU07955.1	PGDFELDHFKMEDLLVTVHQPGGFRPFS~VSIFSCELPQLRKQWLFYDFLSANHMSGSFD
FG01344.1 	PGDFEIEKFKMEDLLLTVHQPGGFRPFS~VSIFSCELPQLRKQWLFYDFLSANHMSGSFD
AN5786.1  	PGDFEIDSFKMSDLLVTVYQPNNFRPFS0----------LRKQWLFYDFLSANMMSGSFD
          	*****:: **:.***:**:**..****.           ******:****.**  *****

MG05135.1 	GSLFTIHPRQIHGVPAGEVG-ASADH-LGEAGAWKKFSRIRIDGLKIDHLNRGVEGPFGW
NCU07955.1	GSLFTIHPRQIHGVSAADNGRHHQDE-FGSPSAWKKFSRLRIDGLKIDHLNRGVEGPFGW
FG01344.1 	GSLFTIHPRQVHGVVPSGNGDPEASVGFGDPKAWKKFSRLRIDGLRIDHLNRGVEGPFGW
AN5786.1  	NSLFTIHPRQTHSFTGAQLGNEAEED--GKPTPWKKHSRIRIDGLNIDHLNRGVQGPFGW
          	.********* *..  .  *    .   *.. .***.**:*****.********:*****

MG05135.1 	IYEGNVDMVADLMFPADEDDSLGKVMSDFYDKMEDAVTKNRYLRILDKNHRGELGERLSE
NCU07955.1	IYEGNVDMIADIMFPADPEEGLSKVVAEFYDKMEEAVTSNRYLKILDGNISPNSRSNTNS
FG01344.1 	IYEGNVDIVADVMFPADTDESITKVMADFYDQLEDIVDTNRHRFIRNIQEQG--------
AN5786.1  	IHEGTVDIVADIMFPADNDESIAKVMADLYDRLEATVTSNRYTEDSNFHDLT--------
          	*:**.**::**:***** ::.: **::::**::*  * .**:    : :           

MG05135.1 	RFSRPATVPMVVLD-ESKGHVSDYTTADADFGPSSPSSRTEEPRTPEAVDEEEPPRYLVM
NCU07955.1	PTLESGPIPESASDIDLTGHHDELPTSEQHFPPLIPPATPKEP-----APIEETPSYLVM
FG01344.1 	--------PALLTS----GHLSNFTSDLLGDKP-----AEEEP-----QTSEEERRYLIM
AN5786.1  	-------------------HIDESKSSDS-------------------------RRFLVT
          	                   * .:  :                              :*: 

MG05135.1 	DLRIHLNDVRAAVPLFTH-DISYISQPLVRPIVAYINAKRTYIPVTCRIVKRTSDFNGSW
NCU07955.1	DLRIHLNDVRAAVPLFNNPHMSYVNQALVRPIVAYINAKRTYIPISCRIVKRLTDFDGAW
FG01344.1 	DLRISMNDVKAAVPLFTK-DMSYVNQALVRPIVAYINAKKTYIPINCRIVKRASDFDGSW
AN5786.1  	DLRLHLNNVRAVVPLFTR-DLSYINNALIRPIVAYINSRRTFIPVNCRLVKRAADFDGSW
          	***: :*:*:*.****.. .:**:.:.*:********:::*:**:.**:*** :**:*:*

MG05135.1 	TLYDSGLMDDMSAE~VYDAFARNVEDQHSRVRRLKKVGFWTLSMVVHALLAGVAGDLI
NCU07955.1	TVWDCGLMDDMSAE~VYRAFARNVEDQQSRVRRFKRVGLWTVSLVVHALLAGVAGDLM
FG01344.1 	SIFDCGLMNDMSAE~TYDAFANDVENQQSRVRRFKKVGFWTLSVAVHALFMGMAGNVV
AN5786.1  	TIYDSGLMDDLSAA0VYDAFARDVVDEQARKRRFKKVGFWSLQLAAQAIFMGMAGNIA
          	:::*.***:*:**  .* ***.:* ::::* **:*:**:*::.:..:*:: *:**::
```
